# Early Experiences Implementing Pre-exposure Prophylaxis (PrEP) for HIV Prevention in San Francisco

**DOI:** 10.1371/journal.pmed.1001613

**Published:** 2014-03-04

**Authors:** Albert Liu, Stephanie Cohen, Stephen Follansbee, Deborah Cohan, Shannon Weber, Darpun Sachdev, Susan Buchbinder

**Affiliations:** 1 Bridge HIV, San Francisco Department of Public Health, San Francisco, California, United States of America; 2 Department of Medicine, University of California, San Francisco, San Francisco, California, United States of America; 3 STD Prevention and Control, San Francisco Department of Public Health, San Francisco, California, United States of America; 4 Kaiser Permanente Medical Center, San Francisco, California, United States of America; 5 Department of Obstetrics, Gynecology, and Reproductive Sciences, University of California, San Francisco, San Francisco, California, United States of America; 6 Department of Family and Community Medicine, University of California, San Francisco, San Francisco, California, United States of America; 7 Department of Epidemiology and Biostatistics, University of California, San Francisco, San Francisco, California, United States of America

## Abstract

Albert Liu and colleagues report early experiences with uptake and delivery of pre-exposure prophylaxis(PrEP)for HIV prevention in three different settings in San Francisco. PrEP can be an important component of a comprehensive HIV prevention program and can complement efforts to increase HIV testing, linkage to care, and early initiation of antiretroviral therapy.

*Please see later in the article for the Editors' Summary*

**Summary Points**

- Pre-exposure prophylaxis (PrEP) has been demonstrated to be safe and efficacious in clinical trials and emtricitabine/tenofovir was approved by the United States Food and Drug Administration for use as PrEP in 2012.
- We report early experiences with PrEP uptake and delivery in three different settings in San Francisco: a PrEP demonstration project in a municipal sexually transmitted diseases (STD) clinic, and two PrEP delivery programs (a private health maintenance organization and an HIV-specific reproductive health program).
- Interest in PrEP is high in San Francisco, particularly among men who have sex with men attending the STD clinic, and it is feasible to incorporate PrEP into busy clinical settings. Key next steps for PrEP implementation include increasing PrEP knowledge; expanding PrEP access; combating PrEP stigma; and optimizing interventions to promote PrEP uptake and adherence while reinforcing risk reduction strategies.
- PrEP can be an important component of a comprehensive HIV prevention program and complement efforts to increase HIV testing, linkage to care, and early initiation of antiretroviral therapy.

## PrEP: Opportunities and Challenges

In July 2012, more than 30 years after the initial HIV case reports, the United States (US) Food and Drug Administration (FDA) approved the first drug for the prevention of sexually acquired HIV infection. On the basis of compelling safety and efficacy data from pre-exposure prophylaxis (PrEP) trials of men who have sex with men (MSM) conducted across four continents and serodifferent heterosexual couples and young men and women in sub-Saharan Africa [1]–[3], the FDA issued the landmark approval of once-daily, co-formulated emtricitabine/tenofovir (FTC/TDF) for HIV prevention in men and women at risk for acquiring HIV infection through sexual exposure. Another recent trial showed PrEP was safe and efficacious in injection drug users in Thailand [4]. PrEP trial results highlight the critical relationship between adherence and efficacy [5], the lack of risk compensation in blinded PrEP trials [2],[6],[7], and the importance of ensuring individuals are HIV-negative prior to initiating PrEP to minimize HIV resistance [8]. Furthermore, modeling studies suggest PrEP can be a cost-effective HIV prevention strategy, particularly if targeted to the highest risk populations [9]–[11].

Although the FDA approval marks an important milestone in HIV prevention, several factors, including negative results from two recently completed PrEP trials among women in Africa [12],[13]; perceived low demand for PrEP [14]–[17]; and concerns about drug adherence [18], spread of antiretroviral resistance [19], medication diversion [20], risk compensation [21], and cost [22] have led to debate on whether and how PrEP should be implemented [23]–[30]. The World Health Organization has issued guidance recommending PrEP demonstration projects be conducted to address these important issues and help determine how PrEP may best be scaled up in different settings to maximize public health impact [31], and a number of PrEP demonstration projects and roll-out programs are being planned or are underway [32]. We describe early experiences with PrEP uptake and delivery in the first year of PrEP implementation post-FDA approval in three different settings in San Francisco, California: an NIH-funded PrEP demonstration project in a municipal STD clinic and two PrEP delivery programs (a managed care organization and a HIV-specific reproductive health clinic). We also propose next steps for the prevention field based on insights learned from our early experiences with PrEP implementation.

## Moving from Efficacy to Effectiveness

On the basis of promising efficacy data from PrEP trials, programs to evaluate PrEP delivery launched in San Francisco, a metropolitan area heavily impacted by HIV, with an HIV prevalence of 23% among MSM [33] and an estimated incidence of 782 cases/100,000 MSM (95% CI 505–1,058) in 2011 [34]. These programs are directly assessing concerns about uptake, adherence, resistance, and risk compensation, as well as models of PrEP delivery in different settings. We describe the core components of these varied programs (see Table 1), present preliminary data on PrEP uptake in these three programs, and summarize lessons learned to date.

**Table 1:** Core components of PrEP delivery programs in San Francisco.

Component	Description
Assess patient as PrEP candidate	• On the basis of local epidemiology, determine risk criteria for delivery of PrEP• Assess for HIV risk at baseline and all follow-up visits• Provide information about risks and benefits of FTC/TDF for PrEP as well as other HIV prevention options• Assess client's interest in starting or continuing PrEP at each visit
Assessment for symptoms of acute HIV infection	• Assess for acute HIV symptoms at baseline and all follow-up visits.• If symptoms concerning for acute HIV, order an individual HIV viral load.• Defer initiation of PrEP until acute infection ruled out.
HIV testing	• Perform HIV testing at baseline and all follow-up visits, at least every 3 months.• Confirm HIV test is negative immediately before dispensing PrEP.• If available, test for acute HIV infection (using 4th generation Ag/Ab test, or pooled/individual HIV RNA) prior to PrEP initiation and at all visits when symptoms of acute HIV infection are reported.• Consider obtaining 4th generation HIV Ag/Ab test at all follow-up visits (window period considerably narrower than current rapid HIV tests).
STD screening (without symptoms)	• For MSM and trans women: syphilis, NAAT-based gonorrhea and chlamydia screening from urine, rectum, and pharynx at baseline and every 3 months or at each encounter for HIV testing• For women: NAAT based gonorrhea and chlamydia screening from vaginal swab (or urine) at baseline and every 6 months
Safety monitoring	• No consensus guidelines exist on optimal frequency or method of kidney function monitoring for patients using FTC/TDF for PrEP (see Table S1).• FTC/TDF should not be dispensed for PrEP if patient has CrCl <60.
Hepatitis B virus (HBV) screen	• At minimum, check hepatitis B surface antigen (HBSAg) at baseline.• If no history of prior vaccination or HBV susceptible, offer HBV vaccine.• If chronically infected, monitor liver function tests closely when stopping FTC/TDF, and consider appropriate medication for HBV treatment.
Reproductive health assessment	• Conduct pregnancy test at baseline and at each follow-up visit.• Evaluate if women are planning to become pregnant, or breast-feeding.• If pregnant, discuss risks/benefits of continuing PrEP with a prenatal provider.• If breastfeeding, discuss risks/benefits of PrEP and continued breastfeeding.
Risk reduction/adherence counseling, side effect management	• Baseline and all follow-up visits.• Optimal strategy for delivering counseling unclear. Counseling approaches for PrEP programs in San Francisco are described in Table S1.
Management of HIV seroconversion	• Patients taking PrEP who have a positive HIV test should be instructed to stop PrEP immediately and be offered post-test counseling and HIV partner services.• Send HIV viral load and genotype and link patient to HIV primary care and treatment in an expedited fashion.

Ag/Ab, antigen/antibody; CrCl, creatinine clearance; FTC/TDF, emtricitabine/tenofovir; MSM, men who have sex with men; NAAT, nucleic-acid amplification test; PrEP, pre-exposure prophylaxis; STD, sexually transmitted disease.

### PrEP in an STD clinic

San Francisco City Clinic (SFCC), the city's municipal STD clinic, offers a range of sexual health services, including HIV and STD screening, diagnosis and treatment, HIV post-exposure prophylaxis (PEP), family planning, and emergency contraception. In 2011, the clinic conducted approximately 19,000 visits with 12,000 patients, of whom 38% were MSM and 1% transgender. HIV incidence among MSM at SFCC is high, with a 2.3% annual seroconversion rate [35]. In October 2012, SFCC began offering PrEP to MSM and transgender women through The Demo Project, a one-year National Institutes of Allergy and Infectious Diseases-funded PrEP demonstration project (Table S1).

From September 2012–2013, 571 individuals have been evaluated at SFCC for PrEP, and 261 participants have enrolled in The Demo Project, for an overall uptake of 49% among potentially eligible clients (Table 2). The most common reasons for declining PrEP include not enough time for study participation (28%), side effect concerns (25%), and no perceived HIV risk (8%). PrEP uptake appears higher among those with prior knowledge about PrEP and those reporting higher risk behaviors. Demand for PrEP has exceeded clinic capacity, with a wait-list of several dozen clients. To date, overall study retention is high; approximately 8% of participants have discontinued PrEP (Table 3). Several participants have expressed anxiety about PrEP access after completing their one-year participation in this project. Project staff are identifying sources of PrEP in the community, linking participants to prevention and care upon project completion.

**Table 2:** PrEP uptake and follow-up in three PrEP delivery programs in San Francisco.

PrEP Uptake Cascade and Follow-up	Date Began Offering PrEP		
	SFCC	Kaiser	BAPAC
	September 2012	April 2012	January 2010
***n*** ** referred/assessed for eligibility**	571	123	15
***n*** ** ineligible** a	40	5	4
***n*** ** potentially eligible**	531	118	11
***n*** ** initiated PrEP**	261	70	7
***n*** ** person-months of follow-up**	1,585	370	24
**Average duration (months) of follow-up (range)**	6.0 (0.3–11.7)	5.3 (0.5–16)	3.4 (1–7)

Data through September 2013.

a Includes medical and behavioral eligibility and program eligibility based on health insurance coverage.

**Table 3:** Reasons for discontinuing PrEP across three PrEP delivery programs in San Francisco.

Reason	Overall *N*
Decreased risk perception	9
Experienced side effects/toxicity	8
Difficulty with medication adherence/monitoring requirements	5
Leaving health plan	4
Concerns about long term side effects	3
Travel	2
Worsening of underlying medical condition	1
Lack of time	1
PrEP stigma	1

PrEP-related stigma (participants feeling stigmatized by others regarding their decision to use PrEP) has deterred some clients from accessing PrEP. Participants in the Demo Project are asked quarterly about social harms related to study participation. Fifteen of 20 social harms reported to date were related to PrEP stigma. Participants reported stigma from peers, who believe that PrEP will lead to increased risk-taking behavior and may divert resources away from HIV-positive people, and medical providers, who are unwilling to prescribe them PrEP or appear judgmental about their decision to use PrEP.

### PrEP in a health maintenance organization

Kaiser Permanente Health Plan provides comprehensive primary and specialty medical care to over 185,000 members in San Francisco. More than 2,500 HIV-positive adults receive care through Kaiser's HIV Care and Prevention Program. Health plan members have a broad spectrum of payor sources for access to Kaiser insurance, including employer-based programs, Medi-Cal, and Healthy San Francisco (a health access program for low income residents of San Francisco) [36]. In May 2012, a PrEP program was initiated in conjunction with other routine HIV prevention strategies (Table S1). Although HIV medication prescribing is restricted to HIV specialists, all adult primary care providers are encouraged to refer interested individuals to the HIV program coordinators, either a pharmacist or nurse, for possible initiation of PrEP. While the number of patients who discuss PrEP with their providers and decide not to pursue a referral is not systematically captured, informal discussions with providers with large panels of MSM suggest that for every person referred, one to three members declined referral after a discussion about PrEP with their provider.

Since the launch of the Kaiser's PrEP program in San Francisco in April 2012, more than half of the 123 men and women referred by both HIV-specialty providers (65%) and non-HIV-specialty providers (35%) have initiated PrEP (table 2). Most common reasons for declining PrEP include decision to use PEP (47%), failure to respond to intake request or initial lab testing (28%), and underlying medical conditions (11%). Of those started, approximately 24% have discontinued FTC/TDF due to a variety of reasons (Table 3).

### PrEP in an HIV-specific reproductive health program

Bay Area Perinatal AIDS Center (BAPAC) is a program of the University of California San Francisco, providing comprehensive preconception management and prenatal care to HIV-positive women and HIV-negative women with HIV-positive male partners (Table S1). BAPAC manages approximately ten to 15 HIV-positive pregnant women and provides preconception counseling for eight to ten patients per year. Potential candidates for PrEP are referred to BAPAC by local providers or are self-referred for consultation on lowering HIV transmission risk during conception and pregnancy. In 2010, BAPAC began offering PrEP to HIV-negative pregnant women who were having sex without a condom with HIV-positive male partners. In 2012, BAPAC expanded the PrEP program to include HIV-uninfected women seeking safer conception options with their HIV-infected male partners. BAPAC has also recently launched the PRO-Men initiative to educate HIV-positive men who have sex with women about their reproductive options and provide preventive services to their HIV-negative female partners, including PrEP. BAPAC provides care for women through six weeks post partum, when women transition to a primary care provider who can continue to prescribe PrEP as needed.

Since 2010, 15 HIV-negative women with HIV-infected male partners have been screened as part of comprehensive HIV prevention counseling at BAPAC for PrEP eligibility. Seven of these women initiated PrEP during prenatal or preconception care, one of whom stopped PrEP after a few days due to nausea and low risk perception given her partner's long term viral suppression. Reasons for not initiating PrEP included lack of insurance coverage, and a combination of low risk perception, concerns about risks to themselves or their babies, and cessation of risk behavior.

While only a few women have initiated PrEP at BAPAC thus far, data suggest HIV-serodifferent couples in the US want to incorporate PrEP as a safer conception option. Between 2006 and 2011, the National Perinatal HIV Hotline and Clinicians' Network took 152 calls regarding conception for serodifferent couples; the volume of calls has increased over time, from ten calls in 2006 to 43 in 2011 [37]. Thirty-four percent of callers sought advice specifically on alternative safer conception interventions such as timed intercourse and PrEP.

## Key Lessons Learned

Lessons learned from three San Francisco PrEP programs can inform future PrEP rollout. First, accurate consumer knowledge about PrEP is a critical first step in PrEP implementation, and prior PrEP awareness was associated with increased uptake in the STD clinic setting. Second, across all three programs, patient risk perception and concern about side effects appear to play an important role in PrEP uptake, adherence, and persistence (continuing PrEP over time) and will need to be addressed to optimize PrEP targeting and implementation. Third, ensuring adequate clinic capacity and sustainable delivery of PrEP is critical to addressing ongoing high demand for PrEP. For time-limited PrEP programs (e.g., Demo Project and BAPAC), it is important to create linkage to ongoing PrEP access after program completion. While this may be a lesser concern for PrEP programs embedded within existing clinical systems (e.g., Kaiser), insurance coverage for PrEP is an important consideration in determining ongoing PrEP access across all programs. Finally, PrEP stigma can pose a barrier to uptake and retention and will need to be addressed among both providers and communities to maximize the impact of PrEP.

## Next Steps for PrEP

On the basis of these lessons learned, we have identified priority next steps to address emerging delivery issues and to maximize PrEP's public health impact. While the examples provided focused on PrEP implementation in the US, several of these concepts and principles may be generalizable to PrEP delivery outside of the US.

### Increase PrEP knowledge

Increasing PrEP knowledge among potential PrEP users is a key step to facilitating PrEP implementation [38]. Community awareness of PrEP appears to be increasing in San Francisco, with PrEP awareness increasing from 20% in 2006 [39] to 44% in 2011 in community-based surveys (Raymond H Fisher, personal communication, September 27, 2013). Increasing PrEP awareness is likely due in part to a range of community engagement activities conducted in collaboration with community partners. For example, co-incident with the initiation of the Demo Project, the San Francisco AIDS Foundation launched a broad PrEP education campaign, including ads on billboards and in transit stations and an informational website (prepfacts.org). Several well-attended public discussion forums were also held in the community. PrEP knowledge among individuals at-risk for HIV has been limited across the US [40] but appears to be slowly increasing after release of iPrEx results [17],[41]. In recent surveys of PrEP knowledge, PrEP awareness ranged from 19% in an online sample of US MSM [17] to 63% among serodifferent couples in San Francisco [15]. Another challenge is that individuals who could benefit from PrEP are often not engaged in care. Collaboration with community partners outside of clinic settings will be critical to reaching at-risk populations, providing PrEP education, and facilitating linkages to PrEP providers [32].

### Expand PrEP access

As interest in PrEP among high seroincidence populations continues to rise in San Francisco, additional PrEP delivery sites in the community are needed. Increasing access to PrEP requires ensuring patients have affordable access to FTC/TDF. Some commercial private insurers are providing coverage for PrEP, although co-pays vary by plan, and some insurers may require prior authorization [42]. The rapidly changing health insurance marketplace, including implementation of the Affordable Care Act, may change PrEP access in as yet unknown ways. Currently, Gilead provides uninsured patients access to FTC/TDF through their medication assistance program [43].

Developing a cadre of skilled providers will also be essential to scaling up PrEP [44]. Many individuals do not feel comfortable discussing risk behaviors with their providers [45],[46], and many providers do not initiate discussion about HIV risk with their patients [47]–[49]. Providers may not have experience prescribing or monitoring patients taking FTC/TDF. Increasing PrEP knowledge among health care providers and identifying and training community providers who are skilled in taking sexual histories and prepared to offer PrEP as part of a comprehensive prevention package will be critical to expanding PrEP access.

PrEP delivery systems will also need to accommodate the increased visit volume associated with scaling up comprehensive PrEP services. PrEP delivery is feasible, but requires staff, time, space, and expertise. A range of models for PrEP delivery have been proposed, including STD clinics [38], primary care clinics [50], and community-based organizations with co-located or linkage to clinical services [51], although each of these settings may face unique challenges in PrEP delivery. For example, while STD clinics serve a population at-risk for HIV infection, most operate on a drop-in or urgent care basis and do not have established systems for providing continuity care or ongoing monitoring (e.g., creatinine testing). Conversely, primary care clinics are experienced with continuity care, but will require approaches to identify patients eligible for PrEP and deliver risk reduction and adherence counseling [50]. Solutions to these issues may be addressed by leveraging existing resources to provide PrEP services, including cross-training of staff (e.g., health educators, pharmacists, nurses) to deliver PrEP counseling [52]. Similar to the movement away from “HIV exceptionalism” and integration of routine HIV testing into primary care settings [53],[54], PrEP delivery will require integration into primary care settings to maximize PrEP coverage of at-risk individuals. Experience and lessons learned from PrEP implementation programs in more specialized settings (e.g., STD/HIV clinics) can be used to develop user-friendly tools (e.g., patient education and counseling materials, checklists for PrEP prescribing) to facilitate primary care providers delivering PrEP in their practices [50].

### Optimize PrEP support

Promoting accurate risk perception and information about potential FTC/TDF side effects and strategies to cope with them will be critical to successful PrEP implementation. Risk assessment tools are currently being developed and validated [55],[56], and several PrEP demonstration projects are evaluating strategies such as text messaging to support adherence and promote retention [32]. Other programs are evaluating the role of real-time pharmacokinetic testing to monitor adherence and trigger enhanced adherence interventions. In The Demo Project, we have developed a one-page handout called “PrEP Basics,” which provides anticipatory guidance regarding PrEP use (see Text S1).

### Combat PrEP stigma

Participants in the Demo Project and other PrEP studies have reported feeling stigmatized by their decision to use PrEP by medical providers, friends, and sex partners. HIV-related stigma and discrimination have a profoundly negative impact on people living with and at risk for HIV [57], acting as deterrents to HIV-testing, serostatus disclosure, and linkage and retention in care [58]. Likewise, PrEP-related stigma could act as an important barrier to PrEP uptake and dissemination. Combating PrEP stigma will require a multi-faceted approach, including social-marketing campaigns, education for health care providers, and a broad recognition of PrEP users as individuals proactively using proven prevention strategies.

## Moving Forward

As the PrEP and HIV prevention fields evolve, several key questions will need to be addressed to optimize PrEP delivery. These issues include determining the optimal target populations for PrEP, standardizing guidelines for initiating and discontinuing PrEP, and evaluating cost-effectiveness. Our early experience with PrEP in San Francisco has illustrated that interest in PrEP is high among populations at risk for HIV infection, and PrEP can be incorporated into busy clinical settings. We believe PrEP can be an important component of comprehensive HIV prevention programs that include efforts to improve HIV testing, linkage to care, and early initiation of antiretroviral therapy.

## Supporting Information

Table S1 **Characteristics of PrEP delivery systems in San Francisco.**
(DOCX)

Text S1 **PrEP basics.**
(DOCX)
