# Supplementary material for: Early Experiences Implementing Pre-exposure Prophylaxis (PrEP) for HIV Prevention in San Francisco
Source: PLoS Med. 2014 Mar 4;11(3):e1001613. doi: 10.1371/journal.pmed.1001613 (PMC3942317; doi:10.1371/journal.pmed.1001613)
Supplement: Table S1 — Characteristics of PrEP delivery systems in San Francisco. (DOCX) [file pmed.1001613.s001.docx]

**Table S1: Characteristics of PrEP delivery systems in San Francisco**

|  | **STD Clinic** | **Health Maintenance Organization (HMO)** | | | **HIV-specific Reproductive Health Program** |
| --- | --- | --- | --- | --- | --- |
| Location | San Francisco City Clinic | Kaiser Permanente | | | BAPAC /PRO-Men: San Francisco General Hospital |
| Target population | 300 sexually active MSM and transgender women, age 18 and over | Adult men and women age 17 and over at perceived or known risk, through sexual or drug use exposure | | | Pregnant women or women wanting to conceive with HIV+ male sex partners |
| Referral/ recruitment | All behaviorally eligible MSM and transwomen attending STD clinic offered participation in PrEP Demonstration Project; also accept referrals from partnering community clinics, CBOs and self-referral | Kaiser patients referred through an electronic consult system with problem “PrEP.” FAQs regarding Kaiser’s PrEP program provided to providers and patients. | | | Referrals from local and regional clinics |
| Behavioral Eligibility / Referral Criteria | Evidence of risk for acquiring HIV-1 infection, including one of the following:   1. Condomless anal sex with 2 or more male or transgender female sex partners during the last 12 months 2. 2 or more episodes of anal sex with at least one HIV+ partner during the last 12 months 3. Sex with a male or transgender female partner and any of the following STDs diagnosed during the last 12 months or at screening: syphilis, rectal gonorrhea or rectal Chlamydia | Evidence of risk for acquiring HIV-1 infection, including one of the following:   1. Frequent initiation of HIV PEP; 2. Risk exposure to multiple partners, with or without concurrent use of recreational drugs 3. A stable, monogamous relationship with an HIV-infected partner (including virologically suppressed) 4. Transfer of prescription from a PrEP study 5. Transfer to Kaiser Permanente Health plan having been prescribed PrEP by a community provider. | | | Evidence of risk for acquiring HIV-1 infection because of sexual relationship with HIV-positive partner(s) (including virologically suppressed). May be pregnant woman or woman considering/planning pregnancy with HIV-positive partner. |
| Medical eligibility | All PrEP programs follow CDC interim guidance on medical eligibility[[22](#_ENREF_22),[23](#_ENREF_23)] (creatinine clearance ≥ 60 ml/min, HIV-negative, check hepatitis B status) | | | | |
| Frequency of visits /refills | Screening visit, enrollment visit, 1 month follow-up, then every 3 months for 1 year | | Screening, and then monthly telephone or electronic follow-up through secure messaging | Screening, and then at least monthly if pregnant | |
| Duration of visits | - Screening: 2.5 hours* - Enrollment and 4 week visit: 1.5 hours - Quarterly visits: 2.5 hours* | | - Screening: 1.5 to 2 hours - Outreach and follow-up through telephone or electronic secure messaging: 0.5 hour/month on average | - Screening: 1-1.5 hours - Follow-up: 0.5-1 hour each | |
| Counseling | - Client-centered, integrated risk reduction and adherence counseling provided by on-site HIV test counselors (part of study staff) | | - Client-directed risk reduction conducted by pharmacist or nurse - Referral to chemical dependency, behavioral medicine, health education as needed | - Client-centered risk reduction counseling - Adherence counseling - PrEP pregnancy and breastfeeding safety data - Increased risk of HIV acquisition during pregnancy - Risks/benefits of breastfeeding if ongoing sexual exposure - Counseling provided by on-site clinicians and clinical social worker | |
| Monitoring of adherence | - Self-reported measures - Pill counts - FTC/TFV levels in dried blood spots and hair | | - Monthly review of self-report - Monthly dispensing of medication with no automatic refill | - Self-report | |
| HIV testing | - Rapid HIV test, 4th generation Ag/Ab test, and HIV RNA pool at screening, enrollment, 1 month, then q3 months | | - HIV antibody testing and HIV RNA at baseline, then HIV antibody testing monthly | - HIV antibody and HIV RNA testing monthly | |
| STI screening | - VDRL - Nucleic acid amplification test (NAAT)-based Gonorrhea and Chlamydia screening from urine, rectum, and pharynx at baseline and q3 months | | - Client directed STI testing/management (oral, rectal, urine (NAAT) GC/CT and syphilis serology) at baseline and monthly | - NAAT based gonorrhea and Chlamydia screening from vaginal swab (or urine) at baseline and q6 months - If pregnant, syphilis serology at baseline and 3^rd^ trimester | |
| Kidney function monitoring | - Serum creatinine, urine dipstick for protein at screening and q3 months | | - Serum creatinine, potassium, phosphate, and urine dipstick at baseline and q 3 months | - Serum creatinine at screening, monthly urine dipstick, creatinine q trimester | |
| HBV screen | - HBSAg at screening - HBV vaccination if not previously vaccinated | | - Confirmation of HBV immunity and vaccination if hepatitis B surface antibody negative | - HBsAg and HBsAb - HBV vaccination if non-immune | |
| Other services provided | - Side effect monitoring | | - Side effect monitoring - Review of HPV and HBV vaccination if <27 years old and initiation if unvaccinated | - Comprehensive prenatal care - Social work/case management - Side-effect monitoring - Enrollment into the Antiretroviral Pregnancy Registry (www.apregistry.com) - Counseling of HIV+ partner: linkage and retention in care; maintain close communication with partner's provider to ensure ART adherence and viral suppression, STI screening/treatment - Monthly support group for HIV+ men who have sex with women (PRO-Men) | |

STD: sexually transmitted disease; CBO: community based organization; HMO: health maintenance organization; BAPAC: Bay Area Perinatal AIDS Center; PRO-men: Positive Reproductive Outcomes for HIV+ Men; MSM: men who have sex with men; HIV+: HIV positive; CDC: Centers for Disease Control and Prevention; FAQ: frequently asked questions; PEP: post-exposure prophylaxis; FTC: emtricitabine; TFV: tenofovir; Ag/Ab: antigen/antibody; GC: gonorrhea; CT: Chlamydia; q: every; HBSAg: hepatitis B surface antigen; HBsAb: hepatitis B surface antibody; RNA: ribonucleic acid; Cr: creatinine

*Study data collection procedures account for approximately 30-45 minutes of each PrEP Demo visit
