# Supplementary material for: Early Experiences Implementing Pre-exposure Prophylaxis (PrEP) for HIV Prevention in San Francisco
Source: PLoS Med. 2014 Mar 4;11(3):e1001613. doi: 10.1371/journal.pmed.1001613 (PMC3942317; doi:10.1371/journal.pmed.1001613)
Supplement: Text S1 — PrEP basics. (DOCX) [file pmed.1001613.s002.docx]

**1. Medication Instructions**

- There are 30-pills of Truvada in each bottle (30-days worth of PrEP).
- Please bring back any leftover pills (in the original bottles) to each visit.
- Store the bottle at room temperature (not in fridge/hot car).
- This medication can be taken with or without food.
- This medication can be taken when drinking alcohol or using drugs.

**2. One Pill Per Day**

- Take 1 pill every day.
- Only studies of daily dosing have shown PrEP to be effective.
- People who use PrEP more consistently have higher levels of protection against HIV.
- You may have to take Truvada for a few days before there is enough in your blood for it to help decrease your chance of getting HIV.
- We have **no** evidence that taking more than one pill a day gives any additional protection. In fact, taking too many can be bad for your health or make you feel sick.
- There are studies going on right now to try to see if less than once a day PrEP would still help to protect people from HIV, but there are no results from these studies yet. Based on what we know right now, we recommend people to take PrEP as close to daily as possible.

**3. Potential Side-effects**

- Some people experience early side effects when taking Truvada for PrEP. This may involve gas, bloating, softer/more frequent stools, or nausea.
- These symptoms are usually mild and go away after the 1st month on PrEP.
- Strategies to deal with stomach related symptoms:
  - take pill with food/snack
  - take pill at night before bedtime
- Contact study staff if you have side effects. We can help.

**4. Sometimes Doses Are Missed**

- People sometimes forget or skip doses. It is not uncommon.
- If you forget a dose just take it when you remember. For example:
  - *If usually take in AM, but realize at 10 pm that you forgot, it’s ok to take 1 pill then and continue with your usual schedule the next day.*

**5. Getting into a Routine**

- Many people find it helpful to take their pills at the same time as something else they regularly do each day (e.g. eating breakfast, brushing teeth).
- Reminders (alarms or seeing the bottle somewhere you look each day) can also help.
- Pill boxes are available if you want to try one.
- When routines are disrupted (e.g., staying out overnight, going on vacation, skipping meals), consider carrying extra pills on you.

**6. Discussing PrEP with Others**

- People sometimes find it helpful to tell friends or family that they are taking PrEP (can help support pill taking).
- Think carefully about who you might want to tell you're taking PrEP (you want it to be someone who will be supportive).
- It’s your personal decision, and you should not feel pressured to tell anyone.

**7. Stopping PrEP**

- Whether or not you want to take PrEP for the full 12-months is your decision.
- If you choose to stop PrEP, please call us and let us know. You do NOT need to be taking PrEP to remain in the study.

**8. Restarting PrEP**

- If you have stopped PrEP for more than 7 days and would like to re-start, please call us and let us know so that we can help you do this safely.
- Getting an HIV test before you re-start PrEP is very important. If you are already infected with HIV and take Truvada, the virus could become resistant to this medication which means that the medication will no longer work for HIV treatment.
- Report any flu-like symptoms or rashes to your provider as they could be symptoms of early HIV infection.

**9. Combining PrEP with other prevention strategies**

- PrEP isn't 100% effective and also doesn't protect against other STDs, so should be combined with other prevention strategies.

**Questions/Concerns**

- Call the clinic if you have any questions or concerns, or if you're going to run out of pills before your next visit. If you have an emergency, call 911 or go to the hospital emergency room.
